# Primary Intradural Extramedullary Spinal Melanoma in the Lower Thoracic Spine

**DOI:** 10.1155/2016/3815280

**Published:** 2016-04-05

**Authors:** Kathrin Hering, Anke Bresch, Donald Lobsien, Wolf Mueller, Rolf-Dieter Kortmann, Clemens Seidel

**Affiliations:** ^1^Department of Radiotherapy and Radiation Oncology, Leipzig University, Liebigstrasse 20, 04103 Leipzig, Germany; ^2^Department of Nuclear Medicine, Leipzig University, Liebigstrasse 20, 04103 Leipzig, Germany; ^3^Department of Neuroradiology, Leipzig University, Liebigstrasse 20, 04103 Leipzig, Germany; ^4^Department of Neuropathology, Leipzig University, Liebigstrasse 20, 04103 Leipzig, Germany

## Abstract

*Background Context.* Up to date, only four cases of primary intradural extramedullary spinal cord melanoma (PIEM) have been reported. No previous reports have described a case of PIEM located in the lower thoracic spine with long-term follow-up.* Purpose*. Demonstrating an unusual, extremely rare case of melanoma manifestation.* Study Design*. Case report.* Methods*. We report a case of a 57-year-old female suffering from increasing lower extremity pain, left-sided paresis, and paraesthesia due to spinal cord compression caused by PIEM in the lower thoracic spine.* Results*. Extensive investigation excluded other possible primary melanoma sites and metastases. For spinal cord decompression, the tumor at level T12 was resected, yet incompletely. Adjuvant radiotherapy was administered two weeks after surgery. The patient was recurrence-free at 104 weeks after radiotherapy but presents with unchanged neurological symptoms.* Conclusion*. Primary intradural extramedullary melanoma (PIEM) is extremely rare and its clinical course is unpredictable.

## 1. Introduction

Here, we report for the first time a case of a 57-year-old female suffering from increasing lower extremity pain, left-sided paresis, and paraesthesia due to spinal cord compression caused by PIEM in the lower thoracic spine. After incomplete resection and adjuvant radiotherapy, the patient is recurrence-free at 104 weeks after radiotherapy but presents with unchanged neurological symptoms.

## 2. Case Report

**Figure 1:**
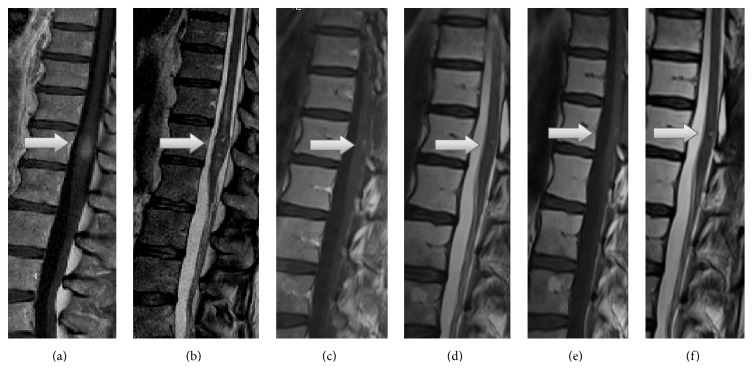
Preoperative sagittal MRI shows the spinal cord tumor at the level of the conus medullaris (arrow). T1-weighted MRI with gadolinium shows high signal intensity relative to that of the cord (a) and T2-weighted MRI shows inhomogenous hypointensive signal (b). Four weeks' follow-up sagittal T1-weighted MRI with gadolinium (c) and T2-weighted MRI (d) and 28 weeks' follow-up T1-weighted MRI with gadolinium (e) and T2-weighted MRI (f) show the residual tumor (arrow).

A 57-year-old female patient was admitted for generalized pain in the lower extremities with mild left-sided distal paresis of the left leg and paraesthesia of the left lower leg and foot progressing for 2 months. Spinal magnetic resonance imaging (MRI) showed an intraspinal mass in the region of the lower thoracic spine at the level of the T12 vertebrae. It was hyperintense on gadolinium enhanced T1-weighted images (Figure 1(a)) and inhomogenous and hypointensive on T2-weighted images (Figure 1(b)). A subsequent MRI scan of the brain and entire spine revealed no other suspect lesions. Due to increasing pain, rapid resection was planned for suspected tumor.

**Figure 2:**
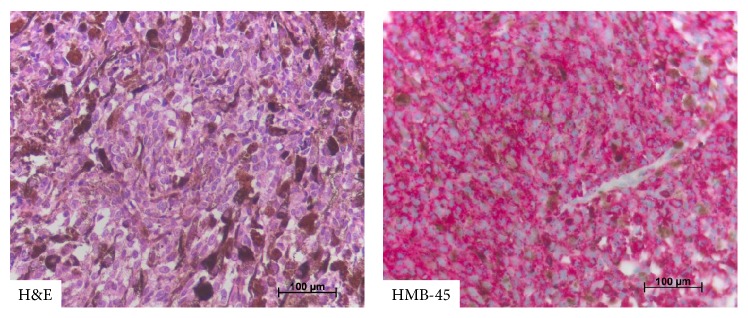
Illustration of the histology (H&E) and immunohistochemistry (HMB-45) of the investigated tumor. Tumor cells are polygonal with prominent nucleoli and partially pigmented (H&E). Tumor cells express the melanoma specific protein HMB-45. Tumor cells were also positive for MELAN-A and S-100 (not shown). Scale bars represent 100 *μ*m; magnification ×400 each.

Intraoperatively, an intradural, black, soft, and hypervascularized neoplasm was encountered which diffusely invaded the dura. Instantaneous surgical section of the tumor confirmed a malignant melanoma. Due to diffuse tumoral infiltration and probable severe postoperative deficits the tumor was incompletely removed with decompression of the neurologic structures (Figures 1(c) and 1(d)). The macroscopic aspect of the lesion was that of a malignant pigmented tumor. Histological examination showed spinal cord tissue with an invading black tumor consisting of tight clusters of tumor cells. The cells were polygonal to spindle shaped with intracytoplasmic brown pigment. Nuclear pleomorphism and mitoses were visible. Immunohistochemistry showed S-100 and HMB45 immunopositivity in tumor cells. They were positive for Melan-A with a mib-labeling index ranging from 5% to 10%.  Figure 2 illustrates histopathology and immunohistochemistry of the investigated tumor. Pyrosequencing of tumor DNA detected no BRAF V600E mutation.

The postoperative phase was uneventful and the neurological state of the patient was unchanged. Parallel to further diagnostics and two weeks after the operation, adjuvant radiotherapy was initiated. With three-dimensional conformal radiotherapy (3D-CRT) we employed a total of 40 Gray in 16 fractions in the area of T11 to L1 vertebrae with no acute toxicity except for mild nausea.

Possible primary melanoma sites and distant melanoma metastases were excluded by computed tomography (CT) scans of the chest, abdomen, and pelvis, esophagogastroduodenoscopy, coloscopy, bone scan, and dermatological, ophthalmological, and gynaecological examination. S-100 was not elevated in the serum. Finally, the diagnosis of primary intradural extramedullary spinal malignant melanoma was established. In the course of the gynaecological examination an early stage invasive ductal breast cancer of the left breast was diagnosed. Surgical treatment was offered to the patient and performed. Adjuvant chemotherapy and radiotherapy were also planned. The patient was regularly in follow-up care. At 8, 20, and 28 weeks after the operation MRI scans of the spinal cord showed a residual lesion with 6 mm length after 20 weeks and 4 mm after 28 weeks. At 15 weeks after surgery, primarily because of the newly diagnosed breast cancer, a whole body 2-deoxy-2-(18F)fluoro-D-glucose positron emission computed tomography (18F-FDG PET/CT) scan was performed to complete staging. Again, no local tumor progress in the spinal cord was visible. The PET/CT revealed two metabolically active skeletal lesions at the third right ridge and at the fifth thoracic vertebral body. A biopsy of both lesions showed no tumor cells. At last follow-up, 104 weeks after radiotherapy, the patient was recurrence-free and showed unchanged neurological symptoms.

## 3. Discussion

All four previously reported cases of primary intradural extramedullary spinal cord melanoma were situated in the cervical spine (Table 1). No report of PIEM in the thoracic spine has been published so far.

It is an extremely rare case, as only 1% of all melanomas located in the central nervous system (CNS) are primary melanomas [5, 6] and primary spinal cord melanoma is even rarer. Clinically, other, more common, differential diagnoses such as primary leptomeningeal melanomatosis, melanoma brain metastases, and primary pigmented tumor of the CNS must be taken into consideration. Our case provides a good illustration of how an extremely rare cause can underlie common neurological symptoms.

The radiological method of choice in the diagnosis of spinal cord melanoma is MRI [7]. Often a lesion in the spinal cord with hyperintensity on native T1-weighted imaging with corresponding hypointense T2 signal is shown. However, MRI findings vary according to the numbers of melanin-containing cells and haemorrhages [6]. Therefore, the distinction between differential diagnoses based solely on their MRI characteristics is difficult. The diagnosis of a melanoma can only be confirmed by histopathological examination. All reported patients with primary intradural spinal cord melanoma underwent surgery as the traditional standard treatment for primary spinal cord melanoma [2, 6]. Due to frequent recurrence of the tumor, radical surgical resection is the first treatment of choice. The role of adjuvant therapy such as radiotherapy or chemotherapy is under debate [5].

There are no exact data for survival rates in patients with primary malignant CNS-melanoma. However, long-term follow-up has shown that primary spinal cord melanoma is generally less malignant than cutaneous melanoma metastatic to the CNS [5, 6].

## 4. Conclusion

Primary intradural extramedullary melanoma (PIEM) is extremely rare and its clinical course is unpredictable.

## Figures and Tables

**Table 1:** Summary of primary intradural extramedullary spinal cord melanoma.

Author	Year	Sex/age	Site	*BRAF* 600E mutation	Operation	Adjuvant therapy	Follow-up	Last state
Mlaiki et al. [1]	2004	M/51	C5–C7	Unknown	Total resection	Unknown	19 weeks	Alive
Kounin et al. [2]	2005	F/41	C2–C4	Unknown	Total resection	None	12 weeks	Alive
Kanatas et al. [3]	2007	F/76	C6-C7	Unknown	Subtotal resection	RT; 30 Gy	24 weeks	Alive
Lee et al. [4]	2010	M/39	C1–C6	Unknown	Total resection	RT; 45 Gy	68 weeks	Alive
Our data	2016	F/57	T12	No	Subtotal resection	RT; 40 Gy	104 weeks	Alive

M: male; F: female; RT: radiation therapy.
